# The waveguiding of sound using lines of resonant holes

**DOI:** 10.1038/s41598-019-47988-7

**Published:** 2019-08-08

**Authors:** G. P. Ward, A. P. Hibbins, J. R. Sambles, J. D. Smith

**Affiliations:** 1 Electromagnetic and Acoustic Materials Group, Department of Physics and Astronomy, University of Exeter, Stocker Road, Devon, EX4 4QL United Kingdom; 2 DSTL, Porton Down, Salisbury, Wiltshire SP4 0JQ United Kingdom

**Keywords:** Surfaces, interfaces and thin films, Acoustics

## Abstract

The dispersion of an acoustic surface wave supported by a line of regularly spaced, open ended holes in an acrylic plate, is characterised by precise measurement of its localised acoustic fields. We illustrate the robust character of this surface wave and show its potential for control of sound by the acoustic waveguiding provided by a ring of regularly spaced holes. A single line of open-ended holes is shown to act as simple acoustic waveguide that can be readily manipulated to control the flow of sound.

## Introduction

Sound is one of nature’s oldest explored phenomena. Thomas Young, famed for his optical two slits experiment, studied in some detail sound’s properties^1^, while, in the nineteenth century Lord Rayleigh’s two volume tome ‘The Theory of Sound’^2^, covered in substantial depth most of this classical field of study. Recently however, there has been a dramatic increase in research around sound interacting with the ever-growing field of metamaterials. The use of metamaterials to control the propagation of surface energy can be traced back to the 1940’s^3^, However it was Ebbesen *et al*.*’s* 1998 work^4^ that is in many ways responsible for the explosion of interest in the use of structured surfaces to generate and control localized surface waves. Following the realization by Pendry *et al*.^5^ that with a simple holey metallic plate it was possible to excite a surface-plasmon-like resonance (the ‘spoof-surface-plasmon’) even when the metal is perfectly conducting, came the reawakening that materials structured on scale lengths of order of or less than the wavelength’s being probed, metamaterials, could provide a wide range of designer-controlled responses to incident waves. From this soon followed the realization of acoustic metamaterials^6,7^, breathing fresh life into this somewhat dormant research field.

The ‘spoof surface plasmon’ is a mode localised to a structured surface, whose fundamental resonant frequency (much lower than the electron plasma frequency) is dictated by the structure. Although, in acoustics, there exists no direct acoustic equivalent to the surface plasmon, there is an analogue to the ‘spoof surface plasmon’: an ‘acoustic-surface-wave’ (ASW). (Other terms are used to label these waves, such as such as ‘leaky guided modes’^8^ or Spoof Surface Acoustic Waves (SSAWs)^9^). Such an ASW is supported by perforated, rigid structures^10^. Using bulk, structured solids, i.e. acoustic metamaterials, a range of novel acoustic phenomena have been explored including Enhanced Acoustic Transmission (EAT)^11–13^, collimation and focusing^9,14^, subwavelength imaging^15,16^, negative refraction^17–21^ and cloaking^22,23^. In this present study we explore the acoustic response of possibly the simplest of all ASW supporting surfaces, a line of holes in an assumed infinitely rigid plate. Each of the identical holes is an acoustic resonant cavity, whose resonant frequency is dictated by the thickness of the plate and is modified by diffractive end effects. Close to the resonant frequency of the holes, the collective response afforded by diffractive coupling of the sound field between these holes, there exists an Eigen mode whose fields are strongly localized at the metamaterial surface^10^. This mode has too much momentum to radiate into free space as plane waves. It is thus a trapped wave, an ASW^10–13^, that decays exponentially away from the structured surface.

Here, a near-field measurement technique provides direct imaging, and hence characterisation, of the acoustic waveguiding provided by a single line of rigid-walled open-hole resonators, for the sake of brevity hereafter we term this an ‘Acoustic Line Mode’ (ALM). Using such a line of equally spaced holes arranged to form a ring we illustrate how this ALM may be directed by design illustrating its potential for novel manipulation of acoustic energy.

**Figure 1:**
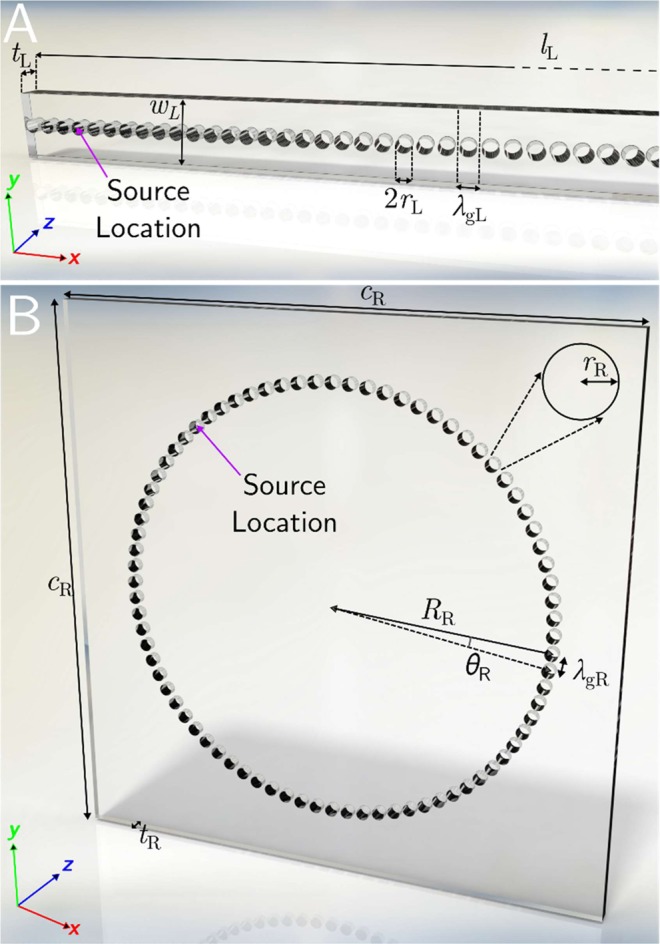
(**A**) Schematic of the 105 hole line (L) sample. The acrylic plate has dimensions *l*_L_ = 840.00 mm (truncated in the figure) and *w*_L_ = 30.00 mm, with thickness (hole depth) *t*_L_ = 9.80 ± 0.10 mm. The periodicity is *λ*_gL_ = 8.00 mm in the x direction. Each hole is of radius *r*_L_ = 3.25 ± 0.005 mm. (**B**) Schematic of the 80 hole ring (R) sample. The acrylic plate has side length *c*_R_ = 29 mm with thickness (hole depth) *t*_R_ = 7.51 ± 0.06 mm. The radius of the ring in which the hole centres are arranged is *R*_R_ = 10.1 ± 0.05 mm. Each of the holes is of radius *r*_R_ = 3.35 ± 0.005 mm, separated by arc *θ*_R_ = 2p/80 giving a centre to centre hole spacing (periodicity) of *λ*_gR_ ≈ 8.00 mm.

The two experimental samples studied are depicted in Fig. 1A,B. Figure 1A is a simple line of 105 equally spaced open-ended holes, laser cut into a plate of acrylic. Figure 1B illustrates the second sample, which is a ring of 80 holes (with periodicity around the ring close to the periodicity in x of the line sample), mechanically drilled into a square plate of acrylic. These structures are designed to support ALMs on their surfaces, which decay away exponentially in z. As will be shown, the ALM on the L-sample is indeed non-radiative. The mode supported by the R-sample is weakly radiative due to the curvature of the line^24^. To achieve excitation of the mode on both samples, the tip of a hollow cone attached to a loud speaker, which approximates a point source, was placed inside one of the holes at the positions indicated in Fig. 1. This loud speaker was driven by a Gaussian-shaped electrical pulse containing a broad range of frequencies (~4–18 kHz), thereby exciting the ALM over a frequency band which includes the expected resonant frequency of each hole. The detector ‘needle’ microphone, mounted on a motorized translation stage and with its 0.5 mm radius tip placed less than 1 mm above the sample surface (which lies in the x-y plane), then records the pulse for a square array of points parallel to the x-y plane covering the area of the sample. This time domain data was then Fourier analysed to provide amplitude and phase for each frequency at each point in space. The dispersion of the surface wave may then be achieved by subsequent spatial Fourier analysis of each individual frequency map.

**Figure 2:**
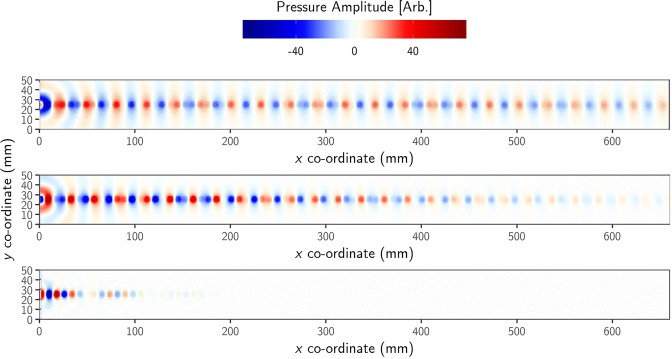
Experimental data of the instantaneous pressure field Δp at three frequencies: top 11 kHz; middle 12 kHz; bottom 13 kHz, measured as a function of x and y coordinates along the surface of the line sample. The point-like source was located at *x* = 0 mm, *y* = 0 mm.

**Figure 3:**
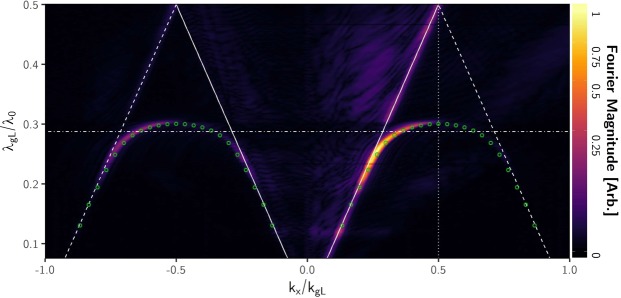
Experimental dispersion diagram for the line sample, obtained from the spatial Fourier transforms of the measured pressure fields on its surface. The magnitude of the Fourier transform is plotted as a function of the ratio of grating periodicity *λ*_*g*L_ to incident wavelength λ_0_ vs the normalized in-plane wavevector, k_x_/k_gL_, along the array surface. The first Brillouin zone is at k_x_/k_gL_ = 0.5. The horizontal dot-dashed line marks the frequency to which Fig. 2 corresponds. The solid white lines represent the ‘sound lines’, the maximum wavevector k_0_ = 2p/l_0_ that a grazing incidence sound wave can possess. The numerically calculated dispersion is represented by the overlaid green circles.

Experimental pressure-field maps illustrating excitation and propagation of the ALM on a single line of holes are presented in Fig. 2. The geometry of the open ended holes are clearly evident in the pressure fields, even at 11 kHz despite the radius *r*_L_ being ~10% of the excitation wavelength of the radiation. By taking the 2D Fourier transform of the pressure field at each frequency component of the excitation pulse, the amplitude of each wave-vector-component (k_x_ and k_y_) is calculated. Then ‘stacking’ in frequency each of these 2D arrays of data and taking a plane through k_y_ = 0 allows visualization of the *f*-*k*_x_ dispersion relation of the modes supported. This procedure gives Fig. 3, the experimentally determined dispersion of the ALM supported by the line sample along the k_x_ direction. Overlaid (green circles) are the Eigenfrequencies of the structure calculated using a finite-element-method (FEM) model. The sound line, k_0_ = 2π/λ_0_, i.e. the dispersion of a plane wave in free space propagating along the surface of the sample, is shown by the white solid line (Acoustic properties for air taken from Cramer^25^).

The ALM is visible in Fig. 3 as a strong feature in the non-radiative regime k_x_ > k_0_, showing that it is indeed trapped and does not radiate from the surface. The mode exists with increasing wave-vector component along the surface until the first Brillouin zone boundary, where it becomes a standing wave with zero group velocity and can no longer be detected with the time-gated pulse technique. (Over most of the frequency range it has a 1/e amplitude decay length of order 10 to 200 mm.) There is a fainter curve of identical shape in the negative half of k-space, i.e. corresponding to a surface wave propagating in the negative x-direction. Because the excitation probe was placed near one end of the sample this dispersion arises from waves reflected from the far end of the sample.

As mentioned, the ALM arises through diffractive near-field coupling between fields in adjacent holes. Given that the fields in the holes are sufficiently well coupled, which is dependent on the frequency, geometry and spacing of the resonators, then ALMs will exist, opening up the potential for bespoke control of acoustic surface energy trapped on a surface. The second sample studied in the work, designed to illustrate this, is comprised of a ring of equally spaced holes with centre points on a ring of radius *R*_R_ (Fig. 1B).

**Figure 4:**
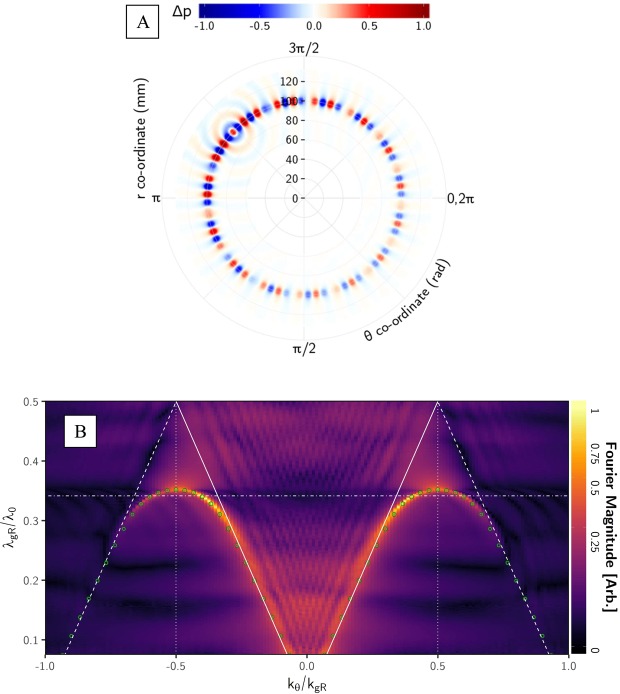
(**A**) Polar plot of experimental data showing instantaneous pressure field Δp at a frequency of 14.625 kHz (l_gR_/l_0_ = 0.341), measured just above the ring sample’s surface. The point-like source was located inside the hole at r ~ 100 mm, θ = 2.3 rad. (**B**) Experimental dispersion diagram for the ring sample, obtained from the spatial Fourier transforms (in polar coordinates) of the measured pressure fields. The magnitude of the Fourier transform is plotted as a function of the ratio of grating periodicity *λ*_gR_ to incident wavelength λ_0_ vs normalized in-plane wavevector k_θ_, along the array surface in the θ direction, along the holes. *λ*_gR_ is defined at the radius *R*_R_, the center of each hole. The solid white lines represent the ‘sound lines’, the maximum wavevector k_0θ_ = 2p/l_q0_ that a grazing incidence sound wave can possess, here in the k_θ_ direction, while the dashed white lines are the diffracted sound lines ± (k_0θ_ + k_gR_), where k_0θ_ and k_gR_ have also been defined at the radius *R*_R._ The horizontal dot-dashed line marks the frequency to which Fig. 4 corresponds. The numerically calculated dispersion is represented by the overlaid green circles.

Figure 4A is the experimentally determined instantaneous pressure field of the ring sample (R) excited by an acoustic pulse, and the data extracted for 14.625 kHz, corresponding to λ_gR_/λ_0_ = 0.341. Similarly to the data from the line (L) sample, (Fig. 2.), it is clear that an ALM is supported by the structure. Following the same procedure described above to produce the data illustrated in Fig. 3, we derive the dispersion of the mode supported by the R-sample (Fig. 4B). The key difference is that the spatial Fourier transforms are performed with an orthogonal grid of polar coordinates rather than Cartesian, allowing representation of the reciprocal lattice as a function of k_θ_ (which is proportional to the angular momentum number^24^) and k_r_. Any plane of this data set taken through k_r_ = 0 illustrates the dispersion. The change of co-ordinates also has important implications for the definition of a trapped surface wave. The angular wavelength λ_θ_ is determined by θ × r, hence the definition of free space wavevector k_0θ_ = 2π/λ_θ0_ is not fixed. This means that at a given frequency, the speed of the curved wavefronts will depend on the radial coordinate r. At some radius, to keep the phase fronts radial, the wavefront would need to travel faster than the speed of sound *c*, hence the wave will always have a radiative component. To make a comparison between the dispersion of the line sample and the ring sample requires that for the ring sample we chose a value for k_0_, using an arbitrary radius to define λ_θ_. To maintain consistency, the radius chosen was *R*_R_ to the center of each hole cavity; at this radius, the periodicity in θ (*λ*_gR_) is equivalent to the periodicity in x (*λ*_gL_) of the line sample (≈8 mm). This has been done in Fig. 4B, where the marked sound lines and Brillouin zones use k_gR_ and k_0θ_ defined accordingly, as well as the grating periodicity λ_gR_.

Here again the ALM is clear in the Fourier transformed data in Fig. 4B with a a dominant feature visible in the regimes|k_θ_|>|k_0θ_|having greater momentum than is possible for a grazing wave, at the radius *R*_R_. With increasing k_θ_, the mode forms a standing wave in θ at its equivalent Brillouin zone asymptote k_θ_/k_gR_ = 0.5, with its decay length along θ decreasing as this asymptote is approached. The overlaid green circles represent the Eigenfrequency solutions from the FEM model. Thus, these ALMs are robust and can indeed follow lines of holes on a surface, provided the local in-plane curvature of the line is not so severe (i.e. small *R*_R_) that the radiative losses become significant.

In conclusion, it is shown that a line of holes acts as a simple acoustic waveguide through the excitation of a trapped acoustic surface wave demonstrated via a high resolution acoustic imaging technique. Using spatial Fourier transforms, the dispersion of the mode supported by these simple hole structures are directly recorded. A single, one-dimensional line of open-ended rigid-walled holes is characterized, where it is found that the fields in the holes couple and support a strong surface wave. Secondly a line of holes is configured into the shape of a ring, where it is shown that waveguiding persists as a strong feature following the ring’s circumference. This type of trapped surface wave is robust to slow arbitrary changes in direction and offers opportunity as a novel method for controlling sound.
